# Supplementary material for: Development and Assessment of a Pooled Serum as Candidate Standard to Measure Influenza A Virus Group 1 Hemagglutinin Stalk-Reactive Antibodies
Source: Vaccines (Basel). 2020 Nov 9;8(4):666. doi: 10.3390/vaccines8040666 (PMC7712758; doi:10.3390/vaccines8040666)
Supplement: Supplementary file 1 [file vaccines-08-00666-s001.pdf]

Supplementary Figure S1

**Nucleotide sequence**
atgaaggcaatactagtagttctgctatatacatttgcaaccgcaaatgcagacacattatgtataggttatcatgcgaacaattcaa<br>cagacactgtagacacagtactagaaaagaatgtaacagtaacacactctgttaaccttctagaagacaagcataacgggaaact<br>atgcaagatcttgaacaaggcccctctcgacctaaggggatgtaccatagaaggttggatcttagggaatcctcaatgcgacctatt<br>gcttggtgatcaaagctggtcatatatagtagaaaggcctactgctcaaaatgggatttgctacccaggagttttgaatgaagtaga<br>agaactgaaggcacttattggatcaggagaaagggtagaaagatttgagatgtttcccaaaagtacatgggcaggggtagacacc<br>agcagtggggtaacaaaagcttgcccttataacagtggttcatctttctacagaaacctcctgtggataataaaaaccaagtcagca<br>gcatatccagtaattaagggaacttacaacaacactggaaatcagccaatcctttacttctggggtgtgcaccatcctcctgacaca<br>aatgagcaaaatactctgtacggttctggtgatcgatacgttaggatggggactgaaagcatgaactttgccaagagtccggaaatt<br>gcagcaagacctgcggtgaacggtcaaagaggcagaattgattattactggtctgttttaaaaccaggggaaaccttgaatgtgga<br>gtctaatggaaatctaatcgccccttggtacgcatacagatttgtcagcacaaataataaaggagccgtcttcaagtcaaatttacca<br>atagagaattgcaatacaacttgtcaaacacccaagggtgctataaacaccagcctcccatttcagaatatacatccgatcacaatt<br>ggaaaatgtccaaaatatgtgaaaagcacaaaattgagactggccacaggattgaggaatatcccgtctattcaatctagaggcct<br>atttggggccattgccggtttcattgaagggggtggacagggatggtagatggatggtacggttatcaccatcaaaatgagcagg<br>ggtcaggatatgcagccgacctgaagagcacacagaatgccattgacgagattactaacaaagtaaattctgttattgaaaagatg<br>aatacacagttcacagcagtaggtaaagagttcaaccacctggaaaaaagaatagagaatttaaataaaaaagttgatgatggtt<br>tcctggacatttggacttacaatgccgaactgttggttctattggaaaatgaaagaactttggactaccacgattcaaatgtgaagaa<br>cttatatgaaaaggtaagaagccagctaaaaaacaatgccaaggaaattggaaacggctgctttgaattttaccacaaatgcgata<br>acacgtgcatggaaagtgtcaaaaatgggacttatgactacccaaaatactcagaggaagcaaaattaaacagagaagaaatag<br>atggggtaaagctggaatcaacaaggatttaccagattttggcgatctattcaactgtcgccagttcattggtactggtagtctccctg<br>ggggcaatcagtttctggatgtgctctaatgggtctctacagtgtagaatatgtatttaa
**Amino acid sequence**
MKAILVVLLYTFATANADTLCIGYHANNSTDTVDTVLEKNVTVTHSVNLLEDKHNGKLCK<br>ILNKAPLDLRGCTIEGWILGNPQCDLLLGDQSWSYIVERPTAQNGICYPGVLNEVEELKALIGSGERVERFE<br>MFPKSTWAGVDTSSGVTKACPYNSGSSFYRNLLWIIKTKSAAYPVIKGTYNNTGNQPILYFWGVHHPPD<br>TNEQNTLYGSGDRYVRMGTESMNFAKSPEIAARPAVNGQRGRIDYYWSVLKPGETLNVESNGNLIAPW<br>YAYRFVSTNNKGAVFKSNLPIENCNTTCQTPKGAINTSLPFQNIHPITIGKCPKYVKSTKLRLATGLRNIPSI<br>QSRGLFGAIAGFIEGGWTGMVDGWYGYHHQNEQGSGYAADLKSTQNAIDEITNKVNSVIEKMNTQFT<br>AVGKEFNHLEKRIENLNKKVDDGFLDIWTYNAELLVLLENERTLDYHDSNVKNLYEKVRSQLKNNAKEIG<br>NGCFEFYHKCDNTCMESVKNGTYDYPKYSEEAKLNREEIDGVKLESTRIYQILAIYSTVASSLVLVVSLGAIS<br>FWMCSNGSLQCRICI*

Supplementary Figure S**1**. Nucleotide (open reading frame) and amino acid sequences corresponding to the chimeric protein cH6/1 used in this study.

Supplementary Figure S2

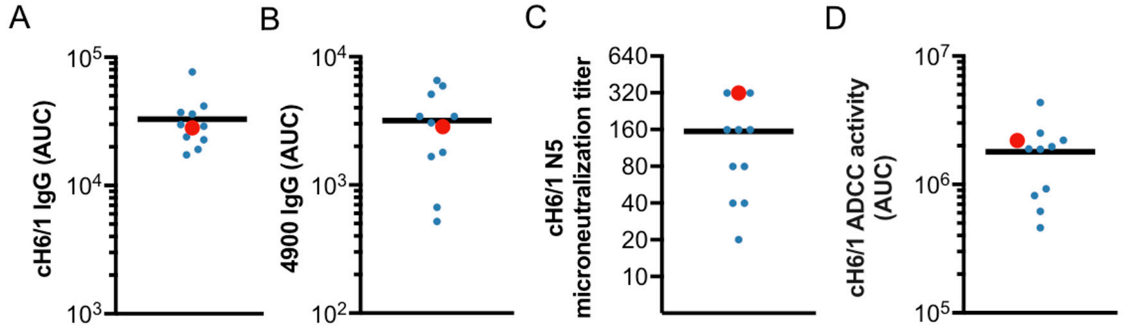

Supplementary Figure S2. The pooled serum displays high levels of stalk-specific antibodies with functional properties. Serum samples from full units and the pooled serum were tested in different binding or functional assays. The standard serum possesses: high levels of cH6/1-specific antibodies (A); high levels of #4900 mini HA-specific antibodies (B); a high neutralization titer (C) and high levels of antibodies with effector functions (D). Dots in A, B and D, represent individual values of area under the curve (AUC), dots in C represent neutralization titers; blue dots represent the full units, the red dot indicates the pooled serum. The arithmetic mean of all values is represented by a black horizontal line.

Supplementary Figure S3

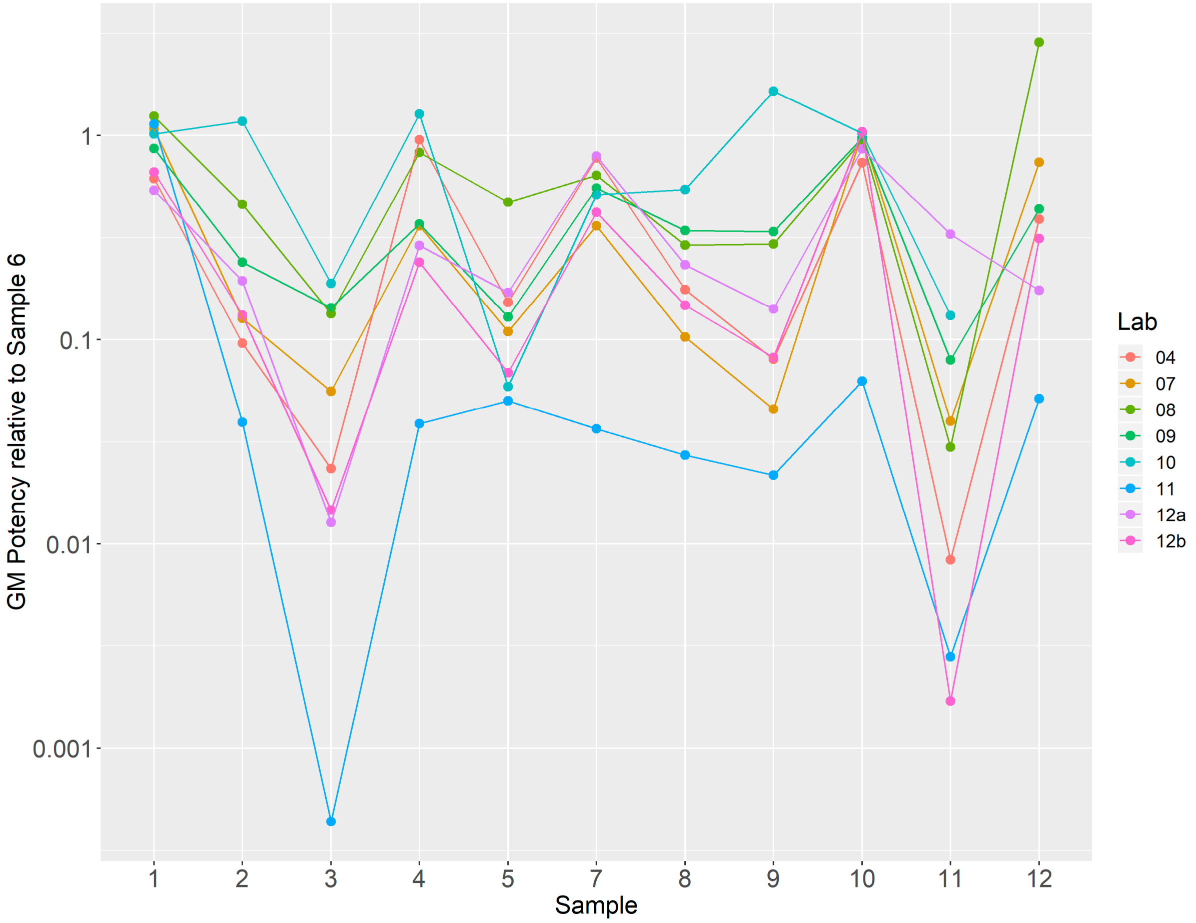

Supplementary Figure S3 Individual laboratory geometric mean potencies relative to candidate standard sample 6. Data shown in Supplementary Table 5 are displayed graphically.

Supplementary Table S1: Sample Panel

Sample #	Sample ID	Response
1	01346	High
2	1985	Intermediate
3	1943	Low
4	01326	High
5	1960	Intermediate
6	Pool	Standard
7	37210	High
8	1978	Intermediate
9	1975	Low
10	Pool	Standard
11	1961	Low
12	03059	High

Supplementary Table S2: Assays performed in different laboratories

	Laboratory Number	2	4	7	8	9	10	11	12a	12b
Binding assays	Assay Type	Magpix high throughput assay	Sandwich ELISA	Indirect ELISA	Competitive ELISA	Bead adsorption ECLIA (Electrochemiluminescence Immunoassay) on the Meso Scale Discovery (MSD) platform	Sandwich ELISA	Direct ELISA	Capture ELISA	Capture ELISA
	Antigen(s)	Multiple ectodomain/head domain HA pairs from group 1 and group 2, including H1N1, H2, H5, H9, H3 and H7	Protein cH9/1HA (chimeric HA with H1 stalk domain from A/Puerto Rico/8/34 and H9 head domain from A/guinea fowl/Hong Kong/WF10/99), 1µg/ml, 100µl/well	Baculovirus expressed cH6/1 recombinant protein	A/California/7/2009 HA and HA1 Purified proteins	H1N1 NC99 stabilized stem (ss) as capture antigen	H1-HA (A/California/2009); H3-HA (A/Switzerland/2013); Group 1 HA stalk (headless H1-HA trimer mini protein); Group 2 HA stalk (headless H3-HA trimer mini protein)	Recombinant chimeric cH6/1 protein	Biotinylated Full-length group 1 HA	Biotinylated mini he adless-HA
	Target being detected	Antibodies binding to ectodmain HA – head domain HA	Group 1 stalk binding IgG	Stalk-reactive IgGs using cH6/1	Indirect measurement of stalk reactive antibodies through pre treatment of serum sample with different concentration of HA and HA1.	H1N1 NC99 Stem-reactive IgGs after pre-adsorption of sample with H1N1 NC99 full length (FL) GLY45	Specific IgG for H1-HA, H3-HA, grp1 stalk, grp2 stalk	Sera of group 1 stalk-reactive IgGs and sera of group 1 stalk-reactive IgAs	Stalk-reactive IgG antibodies using a full-length group 1 HA heterologous to H1	Stalk-reactive IgG antibodies using mini headless-HA trimeric construct of Group 1 stalk
	Titre calculated	MFI	Qutified using human IgG standard curve	Total peak area AUC	Titre calculated based on the signal detected for HA1 and HA2. The HA2 titre is extrapolated from the HA ELISA titre by using the percentage of HA2 signal derived by sera pre-treatment with HA1 and HA protein	AUC	Endpoint titer	AUC (Last X)	ED50	ED50
Functional assays	Assay Type	HA Conformational Change Inhibition Antibody Detection Assay (HCCIA)	Virus neutralization				Microneutralisation assay			
	Antigen(s)	H2/swine/MO/2006, H1/Taiwan/1/86, H1/Brisbane/59/2007	Chimeric virus cH9/1N3, 100TCID50/well				cH6/1 virus			
	Target being detected	Antibodies that can inhibit low pH induced conformational changes of influenza virus hemagglutinin	Group 1 stalk neutralizing antibodies				Stalk reactive Ig using cH6/1 virus			
	Titre calculated	OD ratio of mAb 1/87 4.79 or C179 at pH 4.8 to pH 7.0	The highest dilution factor of sera which gives no hemagglutination with 0.7% turkey RBCs				Inhibitory dilution			

Supplementary Table S3: Sample ED50s excluded from analysis

Lab	Run	Sample	Reason for exclusion
07	4	9	Reduces max:min ratio from 11.1 to 3.1
07	4	11	Reduces max:min ratio from 1.67E+17 to 1.8
10	3	5	Reduces max:min ratio from 828.2 to 3.4
11	1	All	ED50s causing high max:min ratio
11	3	All	ED50s causing high max:min ratio
12a	2	11	Reduces max:min ratio from 250.9 to 2.2

Supplementary Table S4: Geometric mean ED50 estimates

Sample	Lab GM								GM	GCV	Median
	04	07	08*	09	10	11	12a	12b			
1	3481	5933	10756	50726	1796	194787	394	3762	7012	592.1	4847
2	544	701	3978	14027	2075	6760	142	751	1541	352.7	1413
3	133	308	1162	8382	332	75	9	83	230	655.0	221
4	5393	1988	7134	21601	2248	6658	212	1363	3032	301.7	3821
5	864	604	4071	7572	73	8609	124	393	915	512.7	734
6	5666	5509	8647	58581	1762	171438	732	5689	8172	482.7	5677
7	4394	1986	5513	32325	903	6296	580	2384	3243	250.9	3389
8	997	569	2509	20029	959	4679	170	838	1437	324.9	978
9	454	287	2539	19771	2915	3736	103	466	1167	450.9	1503
10	4166	5661	8213	57080	1806	10753	631	5929	5472	272.3	5795
11	47	250	258	4655	232	479	218	10	207	489.9	241
12	2198	4081	24741	25463	N/A	N/A	127	1776	3295	617.5	3139

Supplementary Table S5: Geometric mean potency estimates relative to Sample 6

Sample	Lab GM								GM	GCV	Median
	04	07	08*	09	10	11	12a	12b			
1	0.61	1.08	1.24	0.87	1.02	1.14	0.54	0.66	0.86	37.0	0.94
2	0.10	0.13	0.46	0.24	1.18	0.04	0.19	0.13	0.19	179.5	0.16
3	0.02	0.06	0.13	0.14	0.19	<0.01	0.01	0.01	0.03	627.9	0.04
4	0.95	0.36	0.82	0.37	1.28	0.04	0.29	0.24	0.37	199.0	0.36
5	0.15	0.11	0.47	0.13	0.06	0.05	0.17	0.07	0.12	105.4	0.12
7	0.78	0.36	0.64	0.55	0.51	0.04	0.79	0.42	0.40	171.9	0.53
8	0.18	0.10	0.29	0.34	0.54	0.03	0.23	0.15	0.18	149.1	0.20
9	0.08	0.05	0.29	0.34	1.65	0.02	0.14	0.08	0.14	285.1	0.11
10	0.74	1.03	0.95	0.97	1.03	0.06	0.86	1.04	0.67	162.2	0.96
11	0.01	0.04	0.03	0.08	0.13	<0.01	0.33	<0.01	0.03	541.9	0.03
12	0.39	0.74	2.86	0.43	N/A	N/A	0.17	0.31	0.52	161.2	0.41

*Lab reported results used as ED_{50}s could not be independently calculated
N/A: Excluded due to high inter-assay variability (Max:Min result ratio exceeds 8.0)
GM: Geometric Mean
GCV: Geometric Coefficient of Variation
GCV is shown in red for samples for which GCV was reduced upon normalization relative to sample 6

Supplementary Table S6: Intra-lab variability: Ratios of the maximum and minimum ED50s for each sample in each laboratory; values >8 highlighted

Sample	Laboratory							
	04	07	08	09	10	11	12a	12b
**1**	1.1	1.2	1.0	1.5	1.5	2.9	1.4	1.7
**2**	1.5	1.7	1.3	1.9	1.8	2.1	1.7	1.6
**3**	3.2	2.5	1.2	1.1	5.5	4.3	2.2	1.4
**4**	1.5	1.8	1.3	1.9	1.6	1.9	1.5	1.3
**5**	2.3	2.4	1.3	4.4	3.4	1.8	1.6	1.2
**6**	1.5	1.8	1.6	2.1	3.9	4.3	1.7	1.5
**7**	2.6	3.6	1.2	1.7	2.5	2.7	1.4	2.2
**8**	1.5	3.0	1.2	1.8	1.5	1.7	1.2	1.5
**9**	1.3	3.1	1.5	2.0	3.2	1.6	1.4	1.6
**10**	1.5	2.2	1.6	1.3	3.1	1.4	1.3	1.5
**11**	2.4	1.8	1.6	1.5	3.4	4.5	2.2	3.9
**12**	1.7	2.0	1.3	1.4	15.2	45.8	1.3	1.4

Supplementary Table S7: Intra-lab variability: Ratios of the maximum and minimum potencies for each sample in each laboratory; values >8 highlighted

Sample	Laboratory							
	04	07	08	09	10	11	12a	12b
**1**	1.4	1.7	1.6	1.6	5.2	12.6	1.5	1.2
**2**	1.3	2.3	1.8	2.4	4.8	9.2	1.9	1.1
**3**	3.7	3.1	1.4	1.9	16.8	18.7	2.8	1.9
**4**	1.5	1.9	1.6	1.4	2.8	2.3	1.4	1.2
**5**	1.5	3.1	1.8	5.6	1.9	2.4	1.9	1.5
**7**	2.2	4.7	1.5	2.0	6.9	1.6	1.2	1.7
**8**	1.1	3.2	1.7	2.5	2.6	2.6	1.7	1.2
**9**	1.2	3.2	2.2	2.0	3.7	2.8	1.5	1.3
**10**	1.3	2.0	2.6	1.7	1.9	6.1	1.3	1.2
**11**	1.9	2.2	1.2	2.1	1.6	1.0	1.8	2.9
**12**	1.4	2.0	2.0	1.7	N/A	N/A	1.6	1.1
